# Supplementary material for: Use of family history taking for hereditary neoplastic syndromes screening in primary health care: A systematic review protocol
Source: PLoS One. 2022 Jul 25;17(7):e0271286. doi: 10.1371/journal.pone.0271286 (PMC9312395; doi:10.1371/journal.pone.0271286)
Supplement: Attachment — Submitted filename: Response to Reviewers.docx [file pone.0271286.s002.docx]

**Response to Reviewers**

Vitória, ES, Brazil, May 28th, 2022

*Minor revision*

PONE-D-21-25767

Title : Use of family history taking for Hereditary Neoplastic Syndromes screening in Primary Health Care: a systematic review protocol

Dear Dr. Lopes-Junior,

Thank you for submitting your manuscript to PLOS ONE. After careful consideration, we feel that it has merit but does not fully meet PLOS ONE’s publication criteria as it currently stands. Therefore, we invite you to submit a revised version of the manuscript that addresses the points raised during the review process.

The manuscript has been seen by a reviewer and their comments may be seen below.

The reviewer has provided some minor methodological suggestions to further improve the systematic review.  Could you please revise the manuscript to carefully address the concerns raised?

**Response:** Dear Dr. Lucinda Shen

Thank you for the opportunity to review the manuscript after the reviewers' suggestions and recommendations.

All points were addressed and/or clarified in this new version. In addition, we responded item by item to the questions raised by the reviewers in this letter.
 **Reviewers' comments:**

Reviewers' comments:

Reviewer's Responses to Questions

**Comments to the Author**

1. Does the manuscript provide a valid rationale for the proposed study, with clearly identified and justified research questions?
The research question outlined is expected to address a valid academic problem or topic and contribute to the base of knowledge in the field.

Reviewer #1: Yes

 2. Is the protocol technically sound and planned in a manner that will lead to a meaningful outcome and allow testing the stated hypotheses?
The manuscript should describe the methods in sufficient detail to prevent undisclosed flexibility in the experimental procedure or analysis pipeline, including sufficient outcome-neutral conditions (e.g. necessary controls, absence of floor or ceiling effects) to test the proposed hypotheses and a statistical power analysis where applicable. As there may be aspects of the methodology and analysis which can only be refined once the work is undertaken, authors should outline potential assumptions and explicitly describe what aspects of the proposed analyses, if any, are exploratory.

Reviewer #1: Yes

 3. Is the methodology feasible and described in sufficient detail to allow the work to be replicable?
Descriptions of methods and materials in the protocol should be reported in sufficient detail for another researcher to reproduce all experiments and analyses. The protocol should describe the appropriate controls, sample size calculations, and replication needed to ensure that the data are robust and reproducible.

Reviewer #1: Yes

 4. Have the authors described where all data underlying the findings will be made available when the study is complete?
The [PLOS Data policy](https://journals.plos.org/plosone/s/materials-and-software-sharing) requires authors to make all data underlying the findings described in their manuscript fully available without restriction, with rare exception, at the time of publication. The data should be provided as part of the manuscript or its supporting information, or deposited to a public repository. For example, in addition to summary statistics, the data points behind means, medians and variance measures should be available. If there are restrictions on publicly sharing data—e.g. participant privacy or use of data from a third party—those must be specified.

Reviewer #1: Yes

 5. Is the manuscript presented in an intelligible fashion and written in standard English?
PLOS ONE does not copyedit accepted manuscripts, so the language in submitted articles must be clear, correct, and unambiguous. Any typographical or grammatical errors should be corrected at revision, so please note any specific errors here.

Reviewer #1: Yes

 6. Review Comments to the Author
Please use the space provided to explain your answers to the questions above and, if applicable, provide comments about issues authors must address before this protocol can be accepted for publication. You may also include additional comments for the author, including concerns about research or publication ethics.

You may also provide optional suggestions and comments to authors that they might find helpful in planning their study.

**Reviewer #1:** Main Theme of the manuscript:

In this manuscript, the authors wanted to identify and evaluate the evidence regarding the impact of family history as a genomic technology for Hereditary Neoplastic Syndromes (HNS) screening in Primary health care. My overall impression of the manuscript is written well. The personal and family history is a great resource to estimate the risk of developing cancer in the family members and it is a critical tool for the physicians to predict the possible outcomes and address health maintenance for the patients, especially in the primary health care settings.
**Response:** Thank you so much for your positive feedback and careful review.

**Comments:**

1. It is good to have no restriction on language for collecting the data from the published studies but how authors are going to deal with the data from the non-English origin?

**Response:** Regarding the language, the authors are fluent in English, Portuguese and Spanish. For articles selected in languages other than English, Portuguese and Spanish, the authors will count on the support of Letters Faculty as well as the Graduate Program in Linguistics at the University which are bonded for the translation of articles to be included in this review.

2. Authors also need to address the potential publication and sample size bias.

**Response:** We have added this issue in the paper. "Publication bias will be checked with funnel plots and Egger’s test.

3. At least three reviewers need to independently check each selected article to minimize the bias.

**Response:** Right. We have added this information throughtout the text.

4. Data extraction should be independently cross-checked.

**Response:** We agree with you. All the steps of sthis systematic review will be independently cross-checked. We wrote this in the data extraction section of the paper for clarity, as suggested. Thanks!

5. The different instruments used for collecting family history may hamper the collation of outcomes.

**Response:** Indeed, this is an important point to be considered. Thanks for this timely comment. We have addressed this issue as a possible limitation of this review.

6. There are a few minor typos and grammatical errors. The author must check the grammatical errors thoroughly.

**Response:** The few minor typos and grammatical errors were addressed.
